# Supplementary material for: An Environmentally Sensitive Silk Fibroin/Chitosan Hydrogel and Its Drug Release Behaviors
Source: Polymers (Basel). 2019 Dec 1;11(12):1980. doi: 10.3390/polym11121980 (PMC6960489; doi:10.3390/polym11121980)
Supplement: Supplementary file 1 [file polymers-11-01980-s001.pdf]

Supplement Information for

An environmentally sensitive silk fibroin/chitosan hydrogel and its drug release behaviors

Zhangpeng Xu[1] · Erni Tang[2] · Huijing Zhao[1]*

1 National Engineering Laboratory for Modern Silk, College of Textile and Clothing Engineering, Soochow University, No. 199 Ren'ai Road, Industrial Park, Suzhou 215123, China

2 China Leather and Footwear Industry Research Institute (Jinjiang) Co.,Ltd， No. 150 Wenhua Road, Hongshan Comprehensive District, Jinjiang 362200, China

Correspondence to zhhj@suda.edu.cn

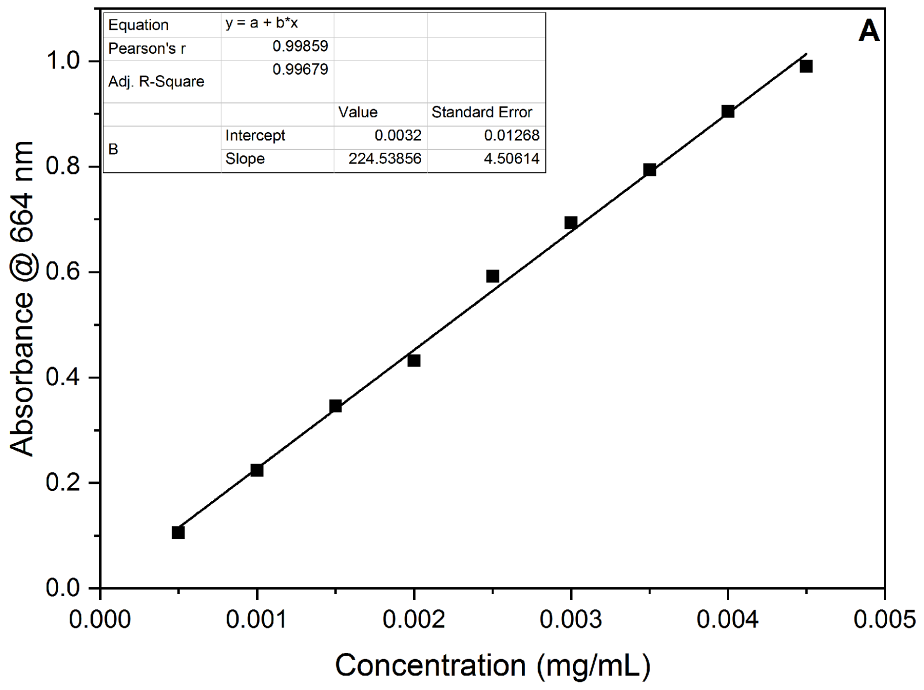
A
Equation
y = a + b*x
Pearson's r
0.99859
Adj. R-Square
0.99679
Value
Standard Error
B
Intercept
0.0032
0.01268
Slope
224.53856
4.50614
Absorbance @ 664 nm
Concentration (mg/mL)

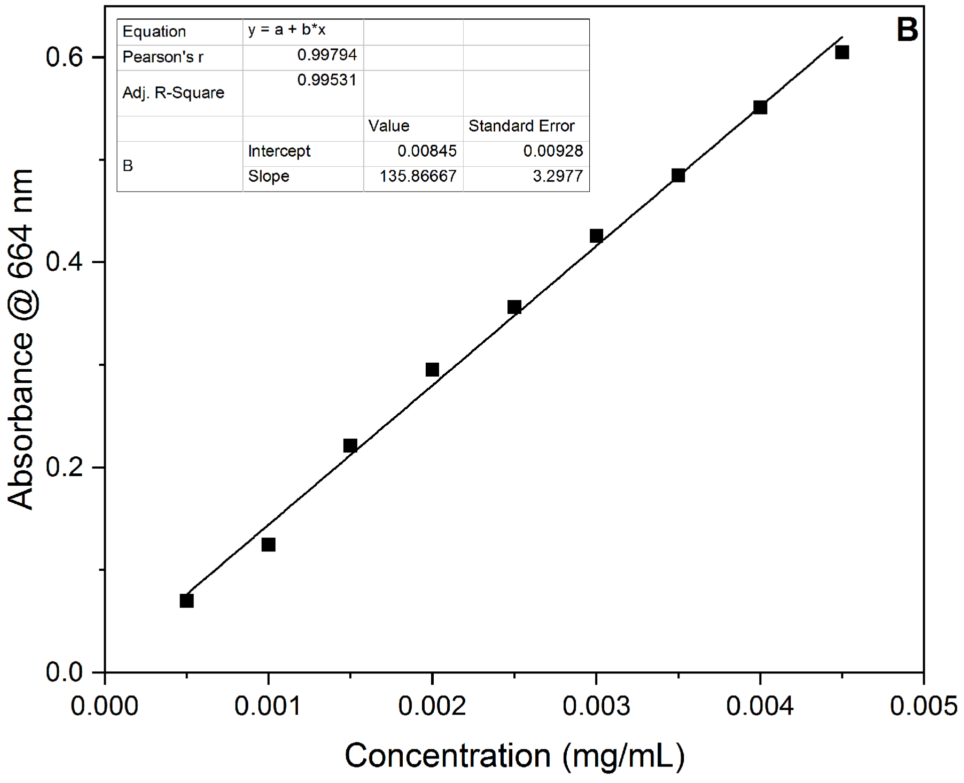
B
Equation
y = a + b*x
Pearson's r
0.99794
Adj. R-Square
0.99531
Value
Standard Error
B
Intercept
0.00845
0.00928
Slope
135.86667
3.2977
Absorbance @ 664 nm
Concentration (mg/mL)

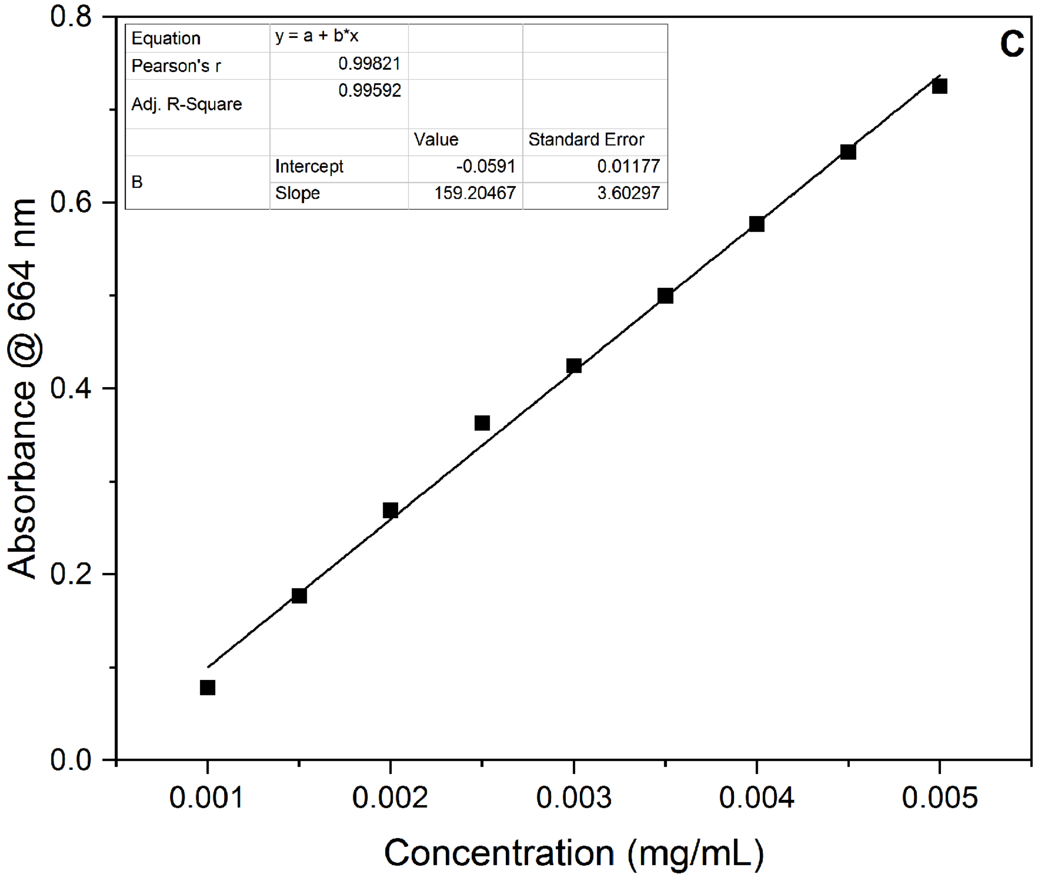

Fig. SI1. (A) Standard curve of MB in deionized water, (B) Standard curve of MB in PBS (pH=7.4, 37℃), (C) Standard curve of MB in PBS (pH=2.2, 37℃).

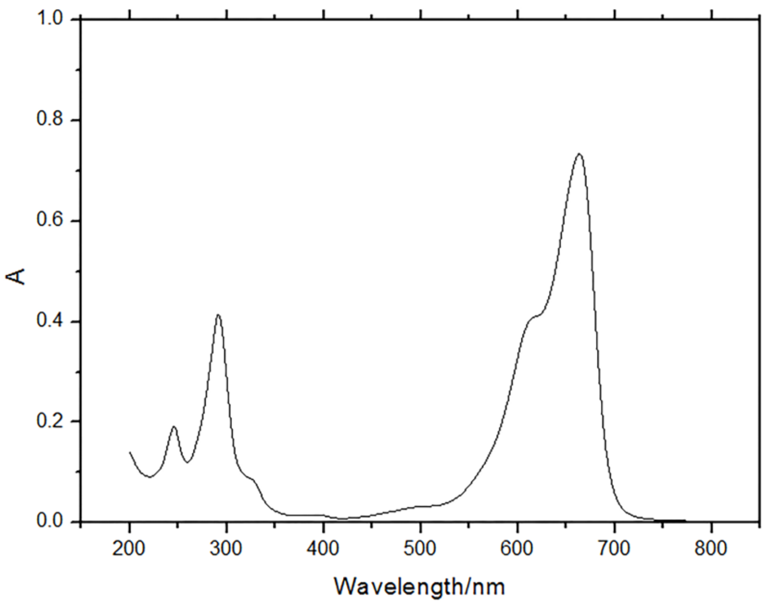

Fig. SI2: UV-visible spectroscopy of MB

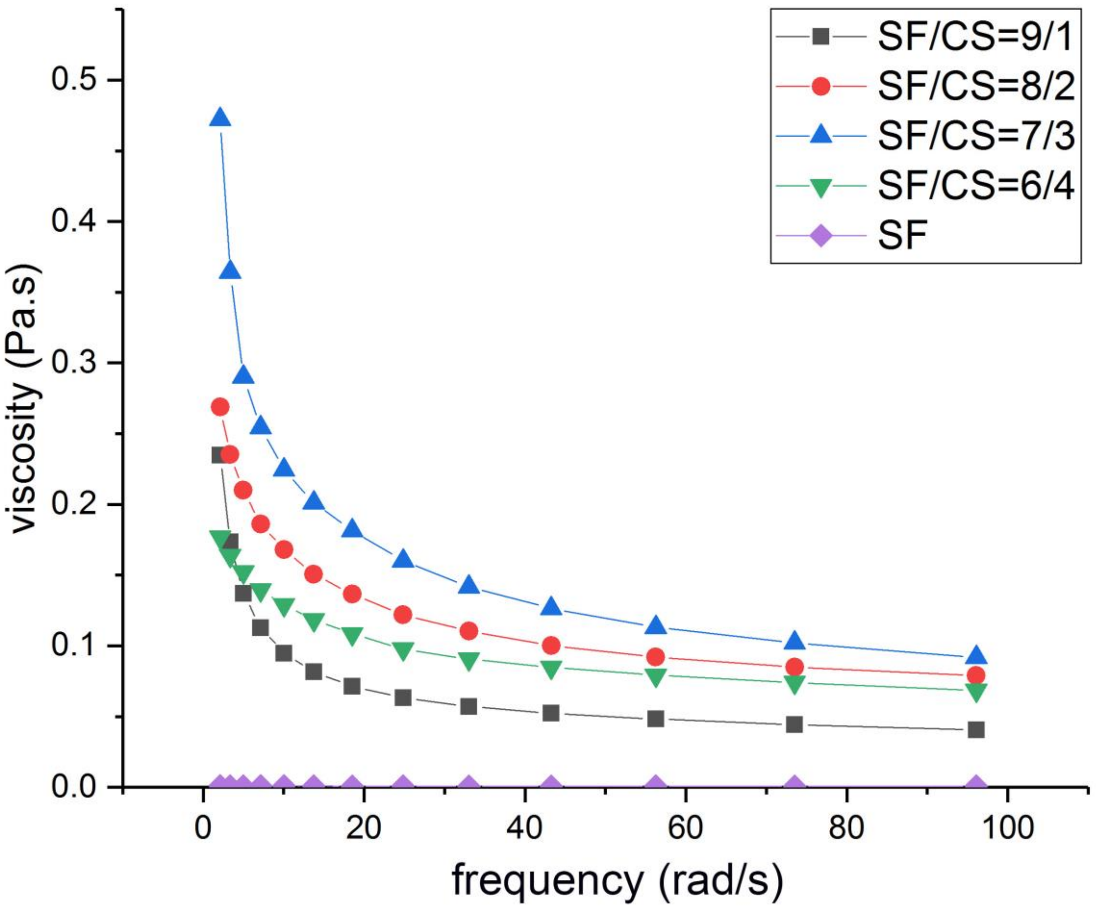

Fig. SI3: The viscosity of mixed solutions.

The dynamic viscosity of mixed solutions was determined by AR-2000 rheometer (TA Instrument, New Castle, USA), fitted with parallel plate (diameter of 20 mm) kept at a gap distance of 1 mm. The shear velocity ranged from 0~100 rad/s.

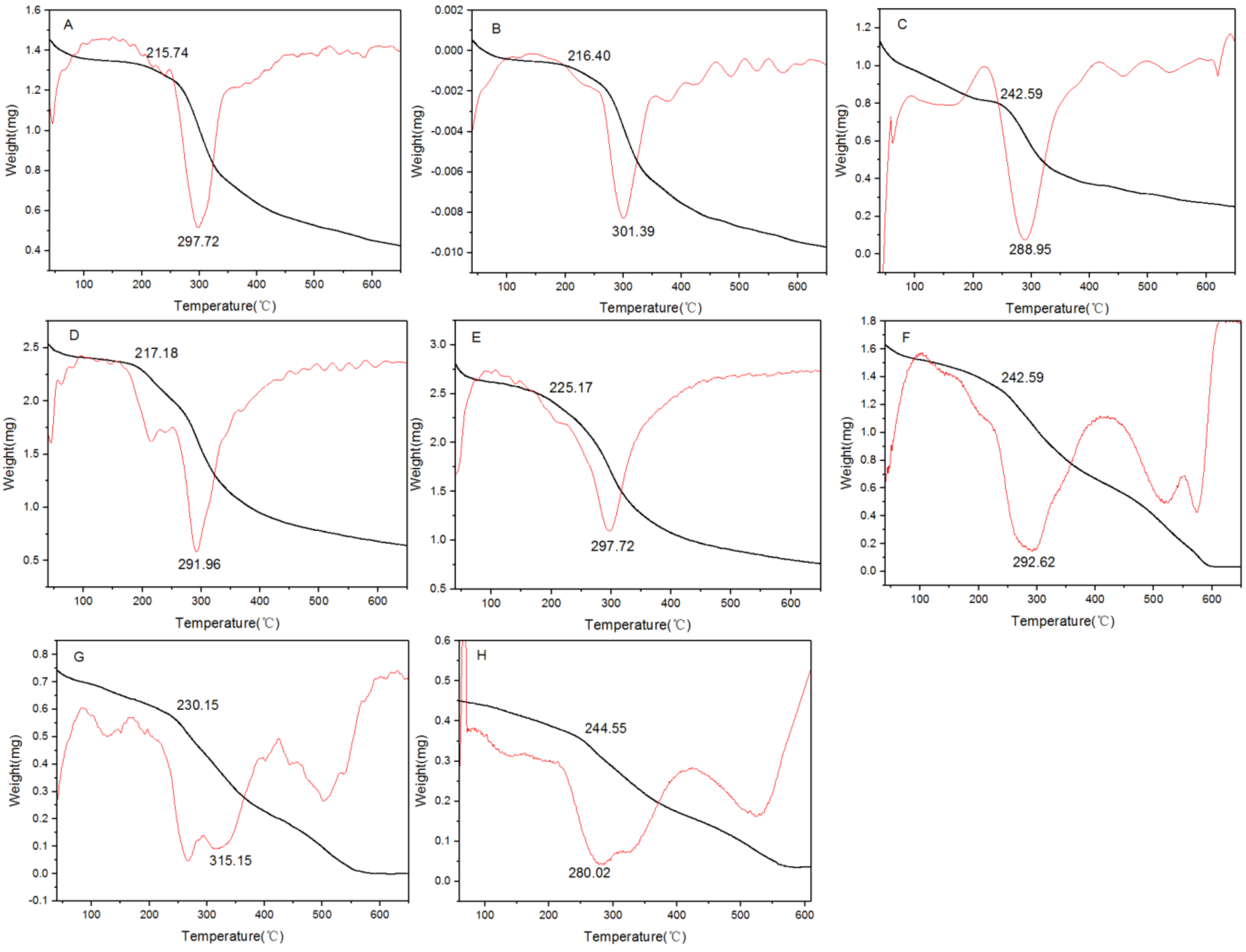

Fig. SI4: The thermograms of hydrogels. (A) SF; (B) NSF; (C) CS; (D) SF/CS=10/0; (E) SF/CS=9/1; (F) SF/CS=8/2; (G) SF/CS= 7/3; (H) SF/CS=6/4
